# Disruption of the Interaction of RAS with PI 3-Kinase Induces Regression of EGFR-Mutant-Driven Lung Cancer

**DOI:** 10.1016/j.celrep.2018.12.003

**Published:** 2018-12-26

**Authors:** Miguel M. Murillo, Sareena Rana, Bradley Spencer-Dene, Emma Nye, Gordon Stamp, Julian Downward

**Affiliations:** 1 Institute of Cancer Research, 237 Fulham Road, London SW3 6JB, UK; 2 Francis Crick Institute, 1 Midland Road, London NW1 1AT, UK

**Keywords:** RAS, KRAS, EGFR, PI3K, PIK3CA, lung cancer, RAC1

## Abstract

RAS family GTPases contribute directly to the regulation of type I phosphoinositide 3-kinases (PI3Ks) via RAS-binding domains in the PI3K catalytic p110 subunits. Disruption of this domain of p110α impairs RAS-mutant-oncogene-driven tumor formation and maintenance. Here, we test the effect of blocking the interaction of RAS with p110α on epidermal growth factor receptor (EGFR)-mutant-driven lung tumorigenesis. Disrupting the RAS-PI3K interaction inhibits activation of both AKT and RAC1 in EGFR-mutant lung cancer cells, leading to reduced growth and survival, and inhibits EGFR-mutant-induced tumor onset and promotes major regression of established tumors in an autochthonous mouse model of EGFR-mutant-induced lung adenocarcinoma. The RAS-PI3K interaction is thus an important signaling node and potential therapeutic target in EGFR-mutant lung cancer, even though RAS oncogenes are not themselves mutated in this setting, suggesting different strategies for tackling tyrosine kinase inhibitor resistance in lung cancer.

## Highlights

• Disrupting the interaction of PI 3-kinase with RAS impairs EGF activation of AKT and RAC
• Mice bred with RAS-binding-domain-defective PI 3-kinase and activated EGFR mutant
• Abrogating RAS binding to PI 3-kinase blocks EGFR-induced lung tumor initiation
• Blocking the RAS-PI 3-kinase interaction induces regression of EGFR-induced tumors

## Graphical Abstract

**Figure:**
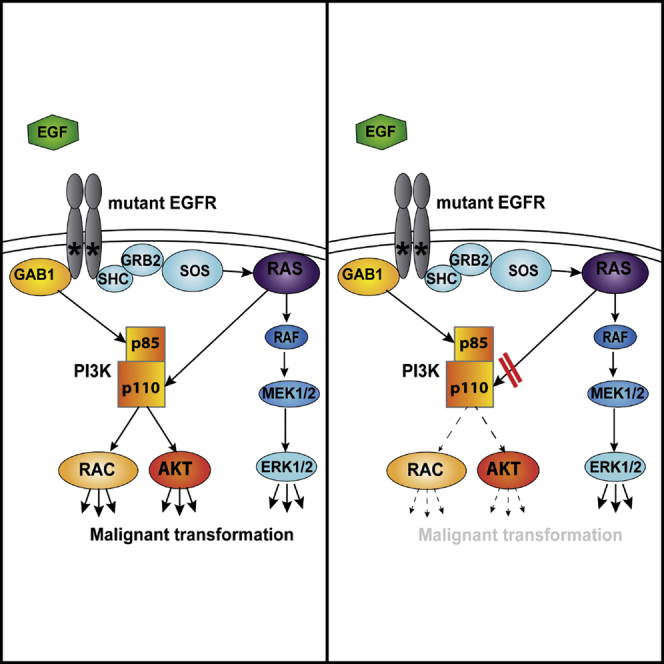

## Teaser

The interaction between RAS and PI 3-kinase is essential for RAS-mutant-induced carcinogenesis. Murillo et al. show that in EGFR-mutant-driven lung cancer, disruption of the interaction of PI 3-kinase with normal RAS proteins blocks tumor initiation and promotes regression of existing tumors, highlighting an unexpected vulnerability of EGFR-driven lung cancer.

## Introduction

The most frequently mutated oncogenes in non-small cell lung cancer (NSCLC) encode the small GTPase KRAS and the epidermal growth factor receptor (EGFR) (Pao and Girard, 2011). The discovery of tumor-driving mutations in EGFR (Lynch et al., 2004, Paez et al., 2004), most commonly in-frame deletions in exon 19 (E746-A750) and a point mutation in exon 21 (L858R), led to the effective use of EGFR-targeted tyrosine kinase inhibitors (TKIs) in NSCLC patients harboring such mutations. However, even though the initial response to these drugs is impressive, secondary mechanisms of resistance eventually lead to disease progression in the majority of patients within a couple of years. Among these mechanisms, the most prevalent is the secondary mutation in EGFR, T790M (Yu et al., 2013). A new generation of tyrosine kinase inhibitors inhibits T790M-mutant EGFR, including osimertinib, which has been approved for clinical use (Jänne et al., 2015, Sequist et al., 2015), but additional mechanisms of resistance have been found in patients treated with these drugs, most notably a C797S mutation in EGFR (Thress et al., 2015). Thus, despite enormous advances in the therapeutic targeting of EGFR, further progress will be needed to achieve effective control or eradication of EGFR-mutant lung cancer.

EGFR controls the activation of several downstream signaling pathways, including RAS small GTPases and the RAF-MEK-ERK and phosphoinositide 3-kinase (PI3K)-AKT pathways. RAS is critical for the activation of the RAF pathway and also contributes directly to activation of the PI3K pathway through direct binding of RAS proteins to a RAS-binding domain (RBD) in the PI3K p110 catalytic subunit (Cox et al., 2014, Fritsch et al., 2013, Rodriguez-Viciana et al., 1994). This interaction is needed for normal development; when the RBD in Pik3ca, the gene encoding p110α, is mutated in the mouse germline so that it cannot bind RAS, transient defects in normal lymphatic development occur as a result of impaired VEGF-C signaling via VEGFR3. Fibroblasts derived from these mice displayed attenuated epidermal growth factor (EGF)-induced signaling to PI3K. Most strikingly, endogenous RAS-mutant-driven tumorigenesis in the lung and the skin is abrogated in these mice with RBD-mutant p110α (Gupta et al., 2007). Moreover, disruption of the RAS-PI3K interaction in preexisting RAS-mutant-driven lung tumors causes partial regression and long-term stabilization (Castellano et al., 2013). This effect is not entirely tumor cell autonomous, as disruption of the interaction of RAS with PI3K p110α only in host tissue also reduces tumor growth and metastasis to some extent by mechanisms that include reduced tumor-induced angiogenesis and alterations in the tumor microenvironment (Murillo et al., 2014).

Since EGFR acts upstream of both RAS and PI3K in lung tumorigenesis, here we explore the effect of disrupting the endogenous RAS-PI3K interaction in a mouse model of EGFR-driven NSCLC, showing that this interaction is required for both tumor onset and maintenance and providing proof of concept that targeting this interaction has potential for treating EGFR-mutant NSCLC, even though RAS genes are not mutated in this setting. The molecular mechanisms underlying the defective signaling of EGFR in the presence of RBD-mutant PI3K involve impaired activation of both AKT and the RHO family GTPase RAC1. It is possible that direct targeting of RAS effector interactions or RAS activation by guanine nucleotide exchange factors may be effective in EGFR-mutant tumors, including those that have become tyrosine kinase inhibitor resistant.

## Results

### RBD-Mutant p110α Expression Inhibits EGF-Induced AKT and RAC Activation

**Figure 1:**
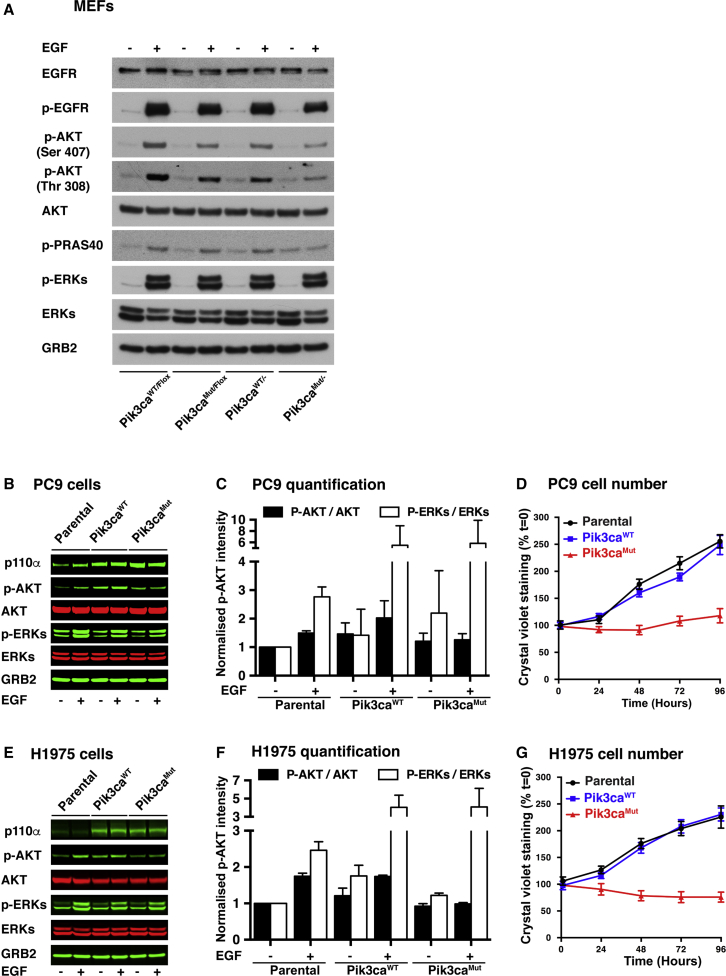
Expression of RAS-Binding Domain-Mutant p110α in EGFR-Mutant Lung Cancer Cells Impairs Growth and Inhibits the Activation of AKT (A) Pik3ca^WT/Flox^ and Pik3ca^MUT/Flox^ MEFs (expressing ROSA26 Cre-ER) were treated overnight with 1 μM tamoxifen or vehicle to generate Pik3ca^WT/−^ and Pik3ca^MUT/−^ MEFs. Cells were starved overnight and stimulated for 10 min with epidermal growth factor (EGF) (10 ng/mL) or vehicle. Proteins were collected and subjected to western blot analysis as indicated. One representative experiment from three is shown. (B and E) PC9 (B) and H1975 (E) EGFR-mutant lung cancer cells were infected with retroviruses encoding Pik3ca^WT^ or Pik3ca^MUT^ or not infected (parental). Cells were then starved and stimulated with EGF (10 ng/mL) or vehicle for 10 min. Proteins were collected and subjected to western blot analysis as indicated. One representative experiment from three is shown. (C) Quantification of the western blots in (B) (n = 3). (F) Quantification of the western blots in (E) (n = 3). (D and G) Cell growth assay by crystal violet staining in PC9 (D) and H1975 (G) cells expressing Pik3ca^WT^, Pik3ca^MUT^ or none (parental). Means of three replicates are shown.

We have previously generated a mouse with an RBD-mutant allele of Pik3ca, the gene encoding PI3K p110α (T208D plus K227A, referred to here as Pik3ca^MUT^) (Gupta et al., 2007). In addition, we have also generated a conditional RAS-binding-domain-mutant p110α mouse model, which has one RBD-mutant allele of Pik3ca (Pik3ca^MUT^) and one floxed, inducibly deletable allele (Pik3ca^Flox^), along with a tamoxifen-activatable ubiquitously expressed Cre recombinase, ROSA26-CreERT2 (Castellano et al., 2013, Murillo et al., 2014). We have previously reported that Pik3ca^MUT/−^ and Pik3ca^MUT/MUT^ mouse embryonic fibroblasts (MEFs) show defective epidermal-growth-factor-induced signaling (Gupta et al., 2007, Murillo et al., 2014). To investigate the mechanism involved in this and also the possible effects of p110α RBD mutation on oncogenic EGFR-induced tumor formation, we tested the effect of endogenous expression of RBD-mutant p110α on EGFR signaling. Analysis of non-recombined or recombined MEFs, with either a Pik3ca^WT^ or a Pik3ca^MUT^ allele, showed that the total levels of EGFR and its tyrosine autophosphorylation in response to EGF were similar (Figure 1A), as were cell-surface EGFR expression levels (Figure S1A). Activation of ERK by EGF was also constant, while induction of AKT activation was decreased, but not abolished, in Pik3ca^MUT/−^ MEFs. Pik3ca^MUT/Flox^ MEFs activate AKT less than Pik3ca^WT/Flox^ MEFs but more than Pik3ca^MUT/−^ MEFs. Activation of AKT downstream of PI3K in response to EGF is thus attenuated, but not abolished, when p110α is unable to bind to endogenous RAS proteins.

In order to extend our observations to the human cancer cell setting, we investigated the effect of impairing p110α RBD function in EGFR-mutant human NSCLC cell lines. We expressed the RBD-mutant form of p110α in PC9 cells (mutant EGFR^ΔE746-A750^), H1975 cells (double-mutant EGFR^L858R-T790M^), and H820 cells (double-mutant EGFR^Del E746-E749-T790M^, plus MET amplification) (Figures 1B, 1E, and S1B–S1D). Even though these cells express the activated EGFR mutant, downstream signaling pathways remain responsive to EGF stimulation. The expression of exogenous MUT-p110α resulted in decreased fold stimulation of the PI3K-AKT pathway upon EGF treatment without significant impairment of ERK pathway activation (Figures 1B, 1C, 1E, 1F, and S1D). Also, cells showed major growth inhibition (Figures 1D, 1G, and S1C), with cells overexpressing a wild-type (WT) form of p110α behaving as parental cells. Thus, some degree of overexpression of a mutant form of p110α that cannot bind to RAS is enough to suppress the activation of the PI3K-AKT pathway in several EGFR-oncogene-driven lung cancer cell lines, including those with additional EGFR T790M resistance mutations and also MET amplification, providing evidence of dominant-negative activity of the RBD-mutant p110α protein over the WT form of the protein.

**Figure 2:**
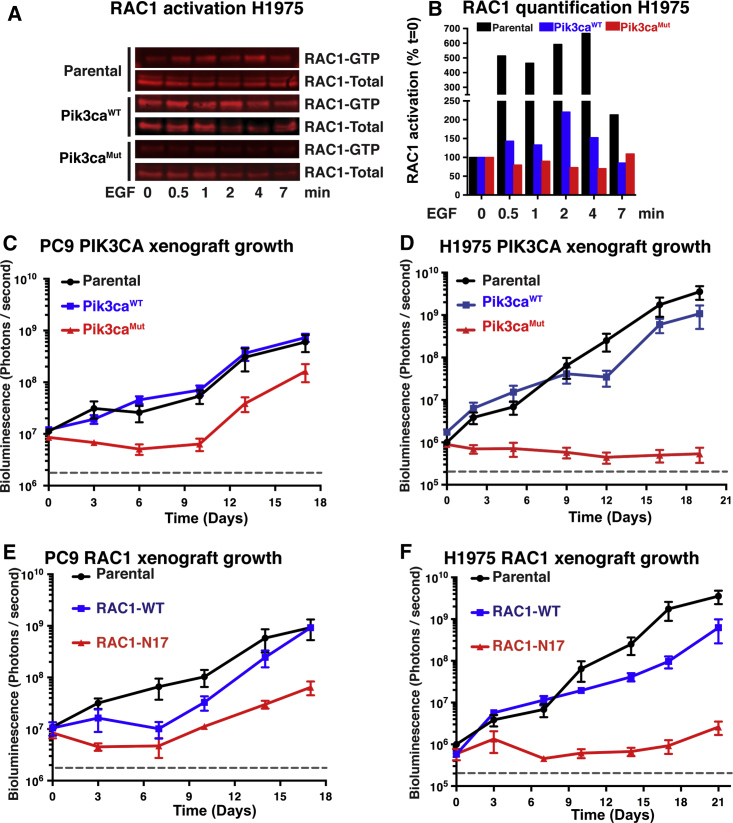
Disrupting the RAS-PI3K Interaction Impairs RAC1 Activation and Reduces Tumor Growth (A) Parental H1975 cells and H1975 cells expressing Pik3ca^WT^ or Pik3ca^MUT^ were starved overnight and stimulated with EGF (10 ng/mL) for the indicated times. Protein extracts were subjected to a RAC1 activation assay. One representative experiment from three is shown. (B) Quantification of western blot in (C). The ratio of RAC1-GTP to total RAC1 is shown, normalized to the value at time 0. (C and D) PC9 (C) and H1975 (D) parental cell lines and cells expressing either Pik3ca^WT^ or Pik3ca^MUT^ were injected into the flanks of nude mice (50,000 cells per mouse). The cells also contain a luciferase-expressing vector. Cells were allowed to grow, and tumor burden was measured by bioluminescence emission for the length of the experiment. (E and F) PC9 (E) and H1975 (F) parental cell lines and cells expressing either a wild-type RAC1 or a dominant-negative version (RAC1^N17^) were injected into the flanks of nude mice (50,000 cells per mouse). The cells also contain a luciferase-expressing vector. Cells were allowed to grow, and tumor burden was measured by bioluminescence emission for the length of the experiment. Means of three replicates are shown (C–F).

Another important signaling pathway downstream of PI3K p110α is the RAC family of RHO GTPases (Campa et al., 2015, Castellano et al., 2016). We explored here whether the expression of RBD-mutant p110α might affect the activation of RAC1, as measured by guanosine triphosphate (GTP) loading, upon EGF stimulation in EGFR-mutant human lung cancer cell lines. While parental H1975 cells activate RAC1 upon EGF stimulation, those expressing RBD-mutant p110α do not, and those exogenously expressing WT p110α show some increased basal RAC1 activation even without EGF addition (Figures 2A and 2B).

### Expression of RBD0-Mutant p110α or Dominant-Negative RAC Inhibits the Growth of EGFR-Mutant Lung Cancer Xenografts

To determine whether impairing the interaction of RAS with PI3K p110α can affect the *in vivo* growth of human EGFR-mutant lung cancer cells in a tumor cell-autonomous manner, we tested the ability of the PC9 and H1975 cell lines described above to form subcutaneous tumors in immunodeficient mice using luciferase expression to monitor tumor growth using bioluminescence. PC9 cells (Figure 2C) and H1975 cells (Figure 2D) expressing MUT-p110α either did not grow or grew only after a long delay, while parental cells and those expressing WT-p110α grew steadily throughout. At the end of the experiment, tumors from WT-p110α or parental PC9 cells were 10-fold larger than tumors from PC9 MUT-p110α cells (Figure S2A), while in H1975 cells, the differential was 10,000-fold (Figures S2B and S2C), demonstrating that expressing an RBD-mutant form of p110α that cannot bind to RAS abrogates the growth of EGFR-driven human NSCLC cell lines *in vivo* in a tumor cell-autonomous manner.

We further extended this analysis to investigate the role played by RAC proteins by infecting H1975 and PC9 cells with a WT or dominant-negative version of RAC1 (RAC1-N17) and monitoring their growth *in vivo* in immunodeficient mice. PC9 cells (Figure 2E) and H1975 cells (Figure 2F) expressing RAC1-N17 either did not grow or grew only after a long delay, while parental cells and those expressing RAC1-WT grew steadily throughout. At the end of the experiment, tumors from RAC1-WT or parental PC9 cells were more than 10-fold larger than tumors from PC9 RAC1-N17 cells (Figure S2D), while in H1975 cells, the differential was over 100-fold (Figures S2E and S2F). RAC1 activation is thus needed for *in vivo* tumor growth of human EGFR-mutant NSCLC cell lines.

### A Mouse Model for Studying the Role of the RAS-PI3K Interaction in EGFR-Mutant-Driven Lung Cancer

To study the effects of disrupting the RAS-PI3K interaction in EGFR-mutant driven lung cancer, we used the conditional RAS-binding-domain-mutant p110α mouse model previously described, which has one RBD-mutant allele of Pik3ca (T208D plus K227A, referred to here as Pik3ca^MUT^) and one floxed, inducibly deletable allele (Pik3ca^Flox^), along with a tamoxifen-activatable ubiquitously expressed Cre recombinase, ROSA26-CreERT2 (Castellano et al., 2013, Murillo et al., 2014). We crossed this strain with mice that inducibly express an EGFR^L858R^-mutant transgene in the lung (Politi et al., 2006) (Figure S3A). These compound mice express a reverse tetracycline-controlled transactivator (rtTA) transgene under the control of the Clara cell secretory protein (CCSP) promoter. Upon tetracycline or doxycycline exposure, the transactivator binds to the tetracycline-responsive promoter of an activated human EGF-receptor-mutant transgene, EGFR^L858R^, inducing its expression and driving lung adenocarcinoma formation. Upon tamoxifen exposure, Cre recombinase becomes active and excises the floxed p110α allele throughout the mouse, leaving only the RBD-mutant allele. Cre-mediated deletion of the floxed p110α allele is essentially complete in various tissues 2 weeks after tamoxifen treatment of mice. Previous work also showed that recombination persisted at this level for at least 8 weeks (Castellano et al., 2013, Murillo et al., 2014), suggesting that there was no appreciable outgrowth of unrecombined cell populations.

We analyzed whether we could tightly control the expression of the EGFR oncogene to induce lung tumor formation using micro-computerized X-ray tomography (micro-CT) *in vivo* and immunohistopathology with an antibody against the EGFR^L858R^ mutant that does not recognize the endogenous, WT EGFR (Figure S3B). Mice containing both the CCSP-rtTA and EGFR^L858R^ genes, but not either singly, express the EGFR mutant only when fed doxycycline-containing food and subsequently develop lung hyperplasia (as seen in the micro-CT images) and discrete tumor nodules (as shown in the histology images).

Given the multifocal hyperplasia characteristic of this EGFR-induced lung tumor model, the use of micro-CT did not readily allow us to monitor the growth of discrete nodules over time (Figure S3B). Therefore, we used micro-CT to compare the amount of air remaining in the lungs after inducing the expression of the oncogene with final tumor burden as measured by histological analysis. A robust inverse correlation between tumor burden and air in the lungs suggests that the latter can be used as a convenient surrogate to follow tumor burden *in vivo* (Figure S3C).

### EGFR-Mutant Lung Tumors Lacking the RAS-PI3K Interaction Show Strongly Delayed Development

**Figure 3:**
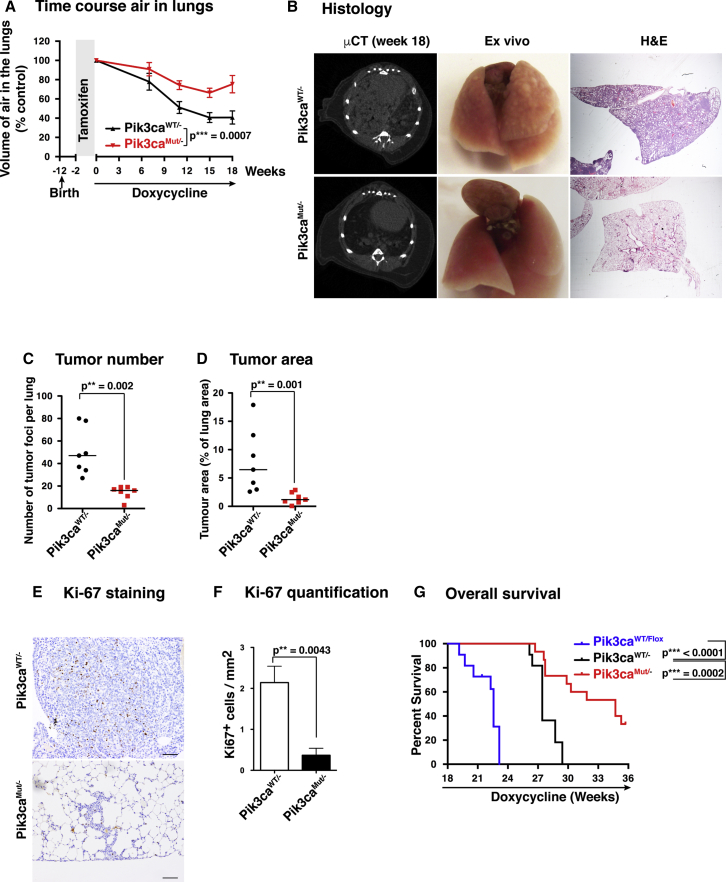
EGFR-Mutant Lung Tumors Lacking the RAS-PI3K Interaction Show Strongly Delayed Development (A) Volume of air remaining in mouse lungs after tamoxifen (2 weeks) and doxycycline diet at different time points (n = 26 for Pik3ca^WT/−^ and n = 21 for Pik3ca^MUT/−^). (B) Representative examples of lungs visualized by micro-CT scan (left), ex-vivo view (middle), and histological sections stained with H&E after 18 weeks of a doxycycline diet. (C) Quantification of the number of tumor foci per lung after 18 weeks of a doxycycline diet from histological samples (n = 7 per group). (D) Quantification of the area of the lung occupied by tumor foci after 18 weeks of a doxycycline diet from histological samples (n = 7 per group). (E) Representative examples of proliferation in tumor samples visualized by Ki-67 staining. Scale bar, 100 μm. (F) Quantification of proliferation by Ki-67 staining (n = 6 per group). (G) Survival assay. The endpoint was dictated by defined welfare severity limits: moderately increased respiratory rate and/or moderately hunched appearance (n = 11 for Pik3ca^WT/Flox^, n = 11 for Pik3ca^WT/−^, and n = 15 for Pik3ca^MUT/−^).

We first analyzed whether early disruption of the RAS-PI3K interaction would affect EGFR^L858R^-driven tumor initiation and formation. Ten-week-old mice were fed tamoxifen-containing pellets for 2 weeks to induce the deletion of the floxed Pik3ca allele. The mice were then fed doxycycline-containing pellets for 18 weeks to induce sustained EGFR^L858R^ oncogene expression, and the amount of air remaining in the lungs was monitored as the tumors grew (Figure 3A). We observed that the air content decreased considerably faster in the lungs of Pik3ca^WT/−^ mice than in those of Pik3ca^MUT/−^ mice. After 18 weeks, Pik3ca^WT/−^ mice had only ∼40% of the initial air content, while Pik3ca^MUT/−^ mice retained ∼75%. Lungs from Pik3ca^WT/−^ mice were highly hyperplastic and contained a large number of tumor nodules, while lungs from Pik3ca^MUT/−^ mice looked normal in size and had a smooth surface, with little or no signs of tumor growth (Figure 3B, central panels). Micro-CT analysis revealed that while Pik3ca^WT/−^ mouse lungs showed a high tumor burden and hyperplasia, the Pik3ca^MUT/−^ lungs were largely normal (Figure 3B, left panels). Histological analysis showed a diffuse hyperplasia with several discrete nodules in Pik3ca^WT/−^ mice, while Pik3ca^MUT/−^ samples showed a normal parenchyma with very small areas of hyperplasia and were almost devoid of tumor nodules (Figure 3B, right panels). The number of tumor foci per lung was much larger in Pik3ca^WT/−^ samples than in Pik3ca^MUT/−^ samples, as was the tumor area (Figures 3C and 3D). Given these differences, we then analyzed proliferation in Pik3ca^WT/−^ nodules compared with Pik3ca^MUT/−^. Ki-67 staining of different samples clearly showed that there was much greater proliferation in Pik3ca^WT/−^ samples than in Pik3ca^MUT/−^ samples (Figures 3E and 3F).

We finally performed a survival experiment comparing these two groups of animals, plus mice that contained two WT copies of p110α (Pik3ca^WT/Flox^ not treated with tamoxifen) (Figure 3G). The results showed that survival of Pik3ca^WT/Flox^ mice was shorter than that of Pik3ca^WT/−^ mice, and survival of the latter group was shorter than that of Pik3ca^MUT/−^ mice, several of which survived beyond the end of the experiment (48 weeks old, with 36 weeks of doxycycline treatment). These results suggest the existence of a gene-dosage effect, as *Pik3ca*^WT/Flox^ mice, with two functional *Pik3ca* alleles, have shorter survival than *Pik3ca*^WT/−^ mice, with one functional *Pik3ca* allele.

We analyzed the degree of recombination in the few tumors that do form in the Pik3ca^MUT/−^ mice, finding that in all cases, these samples contained non-recombined cells, thus indicating that these tumors have escaped the activity of Cre recombinase and continue to express an unrecombined Pik3ca gene (Figure S3D). Altogether, these results show that prior mutation of the RBD of p110α massively diminishes the onset and development of lung tumors in mice driven by the oncogene EGFR^L858R^.

### Removal of the RAS-PI3K Interaction Causes Major Regression of Established EGFR-Mutant Lung Tumors

We next analyzed the effect of disrupting the RAS-PI3K interaction on the continued maintenance of preexisting tumors. For this, Pik3ca^WT/Flox^ and Pik3ca^MUT/Flox^ mice expressing lung-specific doxycycline-inducible EGFR^L858R^ and ubiquitous tamoxifen-inducible Cre recombinase were first fed doxycycline-containing pellets for 11 weeks to induce EGFR^L858R^ oncogene expression and drive tumor formation. A cohort of mice was sacrificed at this point to analyze whether unrecombined Pik3ca^WT/Flox^ and Pik3ca^MUT/Flox^ mice have a similar level of tumor burden. Lungs from both genotypes showed multiple nodules on the surface and a marked hyperplasia (Figure S4A), with similar numbers of tumors and similar overall tumor area (Figures S4B and S4C), although there is a tendency toward slightly lower tumor burden in the Pik3ca^MUT/Flox^ mice, perhaps reflecting the gene-dosage effect observed above. We found no significant differences in the histological grade between Pik3ca^WT/Flox^ and Pik3ca^MUT/Flox^ samples (Figure S4D).

After 11 weeks of doxycycline treatment, we fed the mice tamoxifen-containing pellets for 2 weeks to induce the recombination of the floxed Pik3ca allele, during which time doxycycline was administered by oral gavage. A cohort of mice was sacrificed at different time points to ensure that the EGFR^L858R^ was still expressed and for subsequent histological analysis (Figure S4E). Mice resumed a diet of doxycycline-containing pellets afterward.

**Figure 4:**
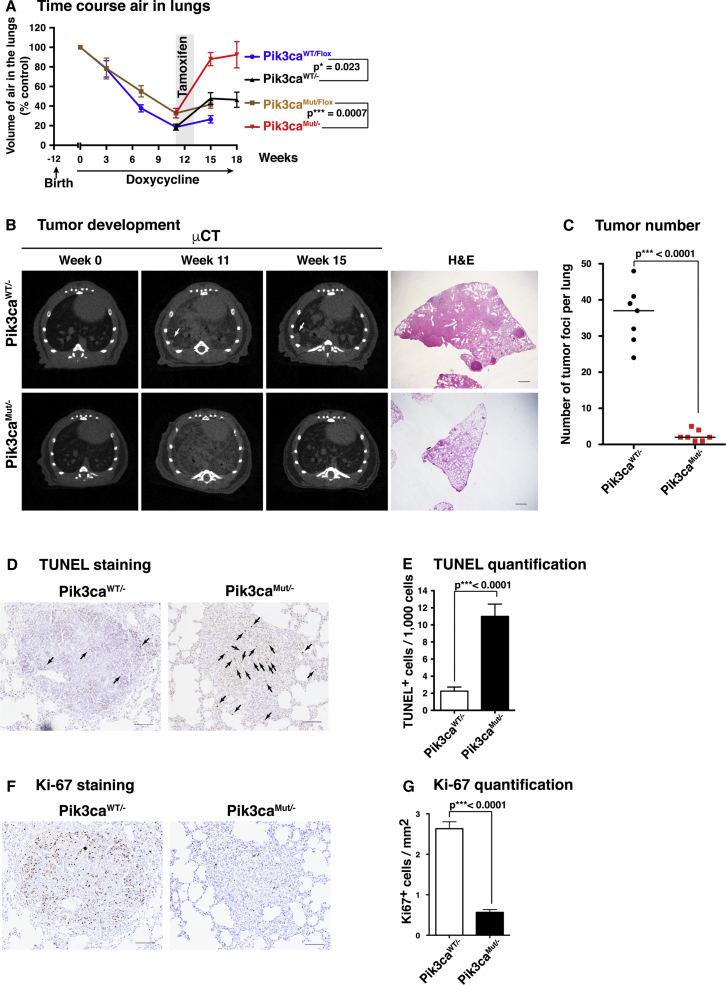
Removal of the RAS-PI3K Interaction Causes Major Regression of Established EGFR-Mutant Lung Tumors (A) Volume of air remaining in the lungs of mice after a doxycycline diet at different time points with (Pik3ca^WT/−^ and Pik3ca^MUT/−^) or without (Pik3ca^WT/Flox^ and Pik3ca^MUT/Flox^) 2 weeks of tamoxifen treatment starting at week 11. Animal numbers are n = 17 (Pik3ca^WT/Flox^) and n = 15 (Pik3ca^MUT/Flox^) from week 0 to week 11 and n = 7 (Pik3ca^WT/Flox^), n = 9 (Pik3ca^MUT/Flox^), n = 10 (Pik3ca^WT/−^), and n = 6 (Pik3ca^MUT/−^) from week 13 to the end of the experiment. (B) Representative examples of micro-CT scans (left three columns) and histological sections stained with H&E (right panel) after 18 weeks of a doxycycline diet. Scale bar, 1 mm. (C) Number of tumor foci (nodules) per lung quantified from histological samples (n = 7 per group). (D) Representative examples of apoptosis visualized by TUNEL staining in histological samples. Scale bar, 100 μm. (E) Quantification of apoptosis by TUNEL staining (n = 20 for each group). (F) Representative examples of proliferation visualized by Ki-67 staining in histological samples. Scale bar, 100 μm. (G) Quantification of proliferation by Ki-67 staining (n = 20 for Pik3ca^WT/−^ and n = 19 for Pik3ca^MUT/−^).

Micro-CT analysis after induction of deletion of the floxed Pik3ca allele revealed a striking increase in the amount of air in the lungs of Pik3ca^MUT/−^ mice (Figure 4A), with neither nodules nor hyperplasia detectable by micro-CT scanning or histology (Figure 4B). By contrast, Pik3ca^WT/−^ mice showed a much smaller increase in lung air content and had continued lung hyperplasia and nodules. Histological analysis 7 weeks after tamoxifen treatment revealed that Pik3ca^WT/−^ lungs samples still had high levels of hyperplasia and discrete nodules, while Pik3ca^MUT/−^ lung samples where virtually devoid of them (Figure 4C). There is a dramatic difference between the level of tumors in Pik3ca^WT/−^ mice compared to Pik3ca^MUT/−^ (Figure 4C), while there is little difference between Pik3ca^WT/Flox^ and Pik3ca^MUT/Flox^ lungs (Figures S4B and S4C), suggesting that a threshold exists in terms of the amount of Pik3ca function needed to support tumor maintenance.

To analyze whether tumor cells in Pik3ca^MUT/−^ mice were dying upon floxed Pik3ca deletion, we isolated lung samples after 5 days of tamoxifen treatment and subjected them to TUNEL and Ki-67 staining (Figures 4D– 4G). Tumors in Pik3ca^MUT/−^ samples showed a very high rate of TUNEL-positive cells, while lower rates were seen in Pik3ca^WT/−^ samples. Conversely, we detected a much higher frequency of Ki-67^+^ cells in Pik3ca^WT/−^ samples compared to Pik3ca^MUT/−^ samples. When TUNEL data were broken down by tumor grade, there were no differences in Pik3ca^WT/−^ nodules according to the tumor grade, but there was a clear increase in TUNEL-positive cells in grade 3 Pik3ca^MUT/−^ nodules compared to grade 2 Pik3ca^MUT/−^ nodules (Figure S4F), suggesting an increasing dependency on the Pik3ca WT allele in more malignant tumors. The tumors in this model do not reach the most invasive state (grade 4), which may require additional deletion of p53; we cannot rule out the possibility that grade 4 tumors might respond differently to Pik3ca RBD mutation.

These results show that disruption of the RAS-PI3K interaction promotes a very major regression of existing EGFR^L858R^-driven lung tumors in mice through both a strong induction of tumor cell apoptosis and a decrease in tumor cell proliferation. This is achieved without obvious toxicity to the mouse, even though the Cre-driven recombination event removing the Pik3ca flox allele occurs throughout the animal.

## Discussion

The p110α catalytic subunit of PI3K is required for signal propagation from RTKs such as EGFR to AKT activation and cellular transformation by oncogenic EGFR mutants (Zhao et al., 2006). In addition, the ability of WT endogenous RAS proteins to interact with p110α via the RBD has been shown to be important for normal EGF signaling to PI3K (Gupta et al., 2007). However, the significance of the interaction of endogenous WT RAS proteins with p110α has not previously been addressed in the context of oncogenic EGFR signaling and in EGFR-mutant-driven cancer models. The development here of a mouse model to determine the effect of disrupting the binding of PI3K p110α to endogenous WT RAS proteins in EGFR-driven lung adenocarcinoma shows that this interaction is critical in tumors that are driven by upstream activators of the RAS pathways and not just those in which RAS is mutationally activated. Mechanistically, EGF-induced activation of both AKT and RAC via PI3K was attenuated in cells expressing RBD-mutant p110α compared to those expressing WT p110α. Blocking RAS’s interaction with PI3K p110α and also activation of RAC1 abrogates the growth of human EGFR-mutant NSCLC in xenografts. This suggests that both RAC and AKT signaling pathways are likely to be important in EGFR-mutant-induced tumorigenesis and that disruption of the RAS-PI3K interaction compromises both events.

The clinical effectiveness of PI3K inhibitors in the treatment of solid tumors remains unclear, and there may be limitations to the ability of these drugs to inhibit oncogenic PI3K signaling in tumors without excessive toxicity to normal tissue. Our experiments here suggest that the RBD of p110α could be a good target for therapy, as blocking its function appears to have limited toxicity in the adult animal while being effective in causing tumor regression. This approach could be effective not only in RAS-mutant-driven cancers but also those driven by mutations in EGFR, and, by extension, possibly in tumors driven by other upstream receptor tyrosine kinases, such as MET and ALK. As the scale of the problem of drug resistance to EGFR tyrosine kinase inhibitors becomes apparent, despite their initial effectiveness, it is likely that other additional approaches will be needed to block signaling though this pathway in order to reduce the likelihood of the evolution of drug resistance (Ramalingam et al., 2018).

There has been much interest recently in the possibility of targeting RAS directly using a number of strategies. Fragment-based screening for RAS inhibitors has identified several compounds that block the interaction of RAS with guanine nucleotide exchange factors (Cox et al., 2014). While there are concerns that inhibitors of RAS activation by exchange factors may be ineffective in tumors with many common activating RAS mutations, the data presented here suggest that such inhibitors could possibly be effective in EGFR-mutant tumors, including those that have become tyrosine kinase inhibitor resistant. This provides a rationale for the further development of existing inhibitors of RAS activation by exchange factors (Burns et al., 2014, Evelyn et al., 2014, Maurer et al., 2012, Patgiri et al., 2011), although the potential toxicity of such an approach remains unclear. In addition, our observations provide support for the more speculative development of inhibitors of the interaction of RAS with PI3K p110α as an approach to treating tumors driven by mutationally activated receptor tyrosine kinases.

## STAR★Methods

### Key Resources Table

**Table:** 

REAGENT or RESOURCE	SOURCE	IDENTIFIER
**Antibodies**		

AKT	Cell Signaling	2920
p-AKT (Thr 308)	Cell Signaling	13038
p-AKT (Ser 473)	Cell Signaling	9271
ERKs	Cell Signaling	9107
p-ERKs	Cell Signaling	9101
p110-alpha	Cell Signaling	4249
GRB2	Cell Signaling	3972
EGFR	Cell Signaling	4267
p-EGFR	Cell Signaling	3777
EGFR L858R	Cell Signaling	3197
p-PRAS40	Cell Signaling	2640
RAC1	Cytoskeleton	ARC03-A

**Chemicals, Peptides, and Recombinant Proteins**		

Tamoxifen	Sigma	H7904
EGF	Sigma	E9644

**Critical Commercial Assays**		

Rac1 Activation Assay	Cytoskeleton	BK035-S
TUNEL	Promega	G7131

**Experimental Models: Organisms/Strains**		

RBD mutant Pik3ca mouse	Jackson Laboratories	B6.129S7(Cg)-Pik3catm1Jdo/J
ROSA26-CRE-ER mouse	Jackson Laboratories	B6.129-Gt(ROSA)26Sor^tm1(cre/ERT2)Tyj^/J
Floxed Pik3ca mouse	Jackson Laboratories	B6N.129- Pik3ca^tm1Jjz^/J
Tetracycline inducible EGFR-L858R	Mouse Repository of the National Cancer Institute	B6;CBA-Tg(tetO-EGFR^∗^L858R)56Hev/Nci
Lung specific reverse tetracycline transactivator CCSP-rtTA mouse	Jackson Laboratories	STOCK Tg(Scgb1a1-rtTA)2Jaw/J

**Recombinant DNA**		

pBABE-puro	Addgene	1764
pLXSN	Clontech	631509

### Contact for Reagent and Resources Sharing

Further information and requests for reagents may be directed to and will be fulfilled by the Lead Contact, Julian Downward (Julian.Downward@Crick.ac.uk).

### Experimental Model and Subject Details

#### Mouse strains and *in vivo* recombination

The Pik3ca^MUT/Flox^ / ROSA26-Cre-ER mouse strain was generated in our laboratory as previously described (Murillo et al., 2014). Targeted allele nomenclature (www.informatics.jax.org) for the RBD mutant Pik3ca mouse was Pik3ca^tm1Jdo^, for the floxed Pik3ca mouse was Pik3ca^tm1Jjz^ and for the ROSA26-CRE-ER mouse was Gt(ROSA)26Sor^tm1(cre/ERT2)Tyj^, with mice being available from the Jackson Laboratory. The tetracycline inducible EGFR-L858R mouse strain was from the Mouse Repository of the National Cancer Institute (allele name Tg(tetO-EGFR^∗^L858R)56Hev). The lung specific reverse tetracycline transactivator CCSP-rtTA mouse (allele name Tg(Scgb1a1-rtTA)2Jaw) was from The Jackson Laboratory. Mice were backcrossed to a C57BL/6J background. The breeding program followed to obtain the final mouse model is depicted in Figure S3A. Experiments were conducted on age matched female mice. *In vivo* recombination of the floxed Pik3ca allele was achieved by feeding mice for 14 days with tamoxifen-containing food pellets (400 ppm; Harlan Teklad) *ad libitum*.

Initial genotyping of all new litters was performed by Transnetyx (Memphis, USA). Assessment of LoxP-p110α recombination was performed by standard PCR using the following primers: forward 5′-CTGTGTAGCCTAGTTTAGAGCAACCATCTA-3′; reverse 5′-CCTCTCTGAAGAGTTCATGTTTGATGGTGA-3′.

All animal experimentation was subject to ethical review by the Francis Crick Animal Welfare and Ethical Review Body and regulation by the UK Home Office project licence 70/8095.

#### Cell culture and stable cell lines

All human cells lines were purchased from ATCC and grown according to ATCC instructions. To generate stable cell lines expressing Luciferase, WT-PIK3CA, MUT-PIK3CA, WT-RAC1 and N17-RAC1, retrovirus containing the indicated genes were generated in Phoenix cells using Lipofectamine 2000 following manufacture instructions. Retrovirus were used to infect cell lines followed by a selection period using a standard protocol.

### Method Details

#### Micro-CT

Mice were anesthetized with 2% isoflurane oxygen gas, and images of the lung region were acquired on a SkyScan 1176 micro-CT analyzer. Data were sorted using Tsort (SkyScan) and reconstructed using the NRecon program (SkyScan). Reconstructed data were subsequently imaged using DataViewer and CTAn programs. The volume of air in the lungs was quantified using CTAn as per manufacturer’s instructions (SkyScan).

#### Tumor burden and bioluminescence

Tumor burden was measured in H&E-stained tissue sections or by bioluminescence. Quantification on H&E samples was performed using NIKON NIS-Elements software according to the manufacturer’s instructions. For bioluminescence, PC9 and H1975 cells were infected with retroviruses containing a pBabe-Luciferase. This vector was engineered by cloning a Luciferase gene containing a poly-A tail in the Bamh1-Sal1 restriction sites of a pBabe-Blastocidine empty vector. Bioluminescence was measured using an IVIS Spectrum Pre-Clinical *In Vivo* Imaging System according to the manufacturer’s instructions. Cell lines were screened periodically to ensure that the levels of luciferase were stable.

#### Histopathological analysis

Tissue samples were fixed overnight in 10% formalin and subsequently stored in 70% ethanol. Samples were then embedded in paraffin and sectioned. Sections were stained for Haematoxylin and Eosin, Ki-67 and TUNEL (Promega G7131) following standard methods or manufacturer instructions. For EGFR-L858R staining (Cell Signaling antibody 3197) sections were microwaved for 15 minutes in 1 mM EDTA pH = 8, followed by an overnight incubation with the antibody at a 1:100 dilution. Scale bars shown are 100μm.

#### Engineering of cDNA constructs

cDNAs for Luciferase, Rac1 and Rac1-N17 were subcloned into a retroviral pBabe vector (Addgene 1764). cDNAs for pic3ca-WT and pic3ca Mutant were subcloned into a retroviral pLXSN vector (Clontech 631509).

### Quantification and Statistical Analysis

All data groups were subjected to a normality test (Shapiro-Wilks). When all data groups within an experiment were normally distributed, a two-tailed unpaired Student’s test was performed to determine differences, and data was represented as means ± SEM. When any group within an experiment was not normally distributed, a non-parametric Mann-Whitney U test was used, and the median was displayed (Figures 3C, 3D, 4C, S2, S4B, and S4C). Figure 3G statistics were generated using a Kaplan-Meir estimator.

## Supplemental Information

Document S1. Figures S1–S4

Document S2. Article plus Supplemental Information
