# Supplementary material for: Disruption of the Interaction of RAS with PI 3-Kinase Induces Regression of EGFR-Mutant-Driven Lung Cancer
Source: Cell Rep. 2018 Dec 26;25(13):3545–3553.e2. doi: 10.1016/j.celrep.2018.12.003 (PMC6315106; doi:10.1016/j.celrep.2018.12.003)
Supplement: Document S1. Figures S1–S4 [file mmc1.pdf]

Cell Reports, Volume *25*

Supplemental Information

Disruption of the Interaction of RAS with PI 3-Kinase Induces Regression of EGFR-Mutant-Driven Lung Cancer

Miguel M. Murillo, Sareena Rana, Bradley Spencer-Dene, Emma Nye, Gordon Stamp, and Julian Downward

SUPPLEMENTARY FIGURES

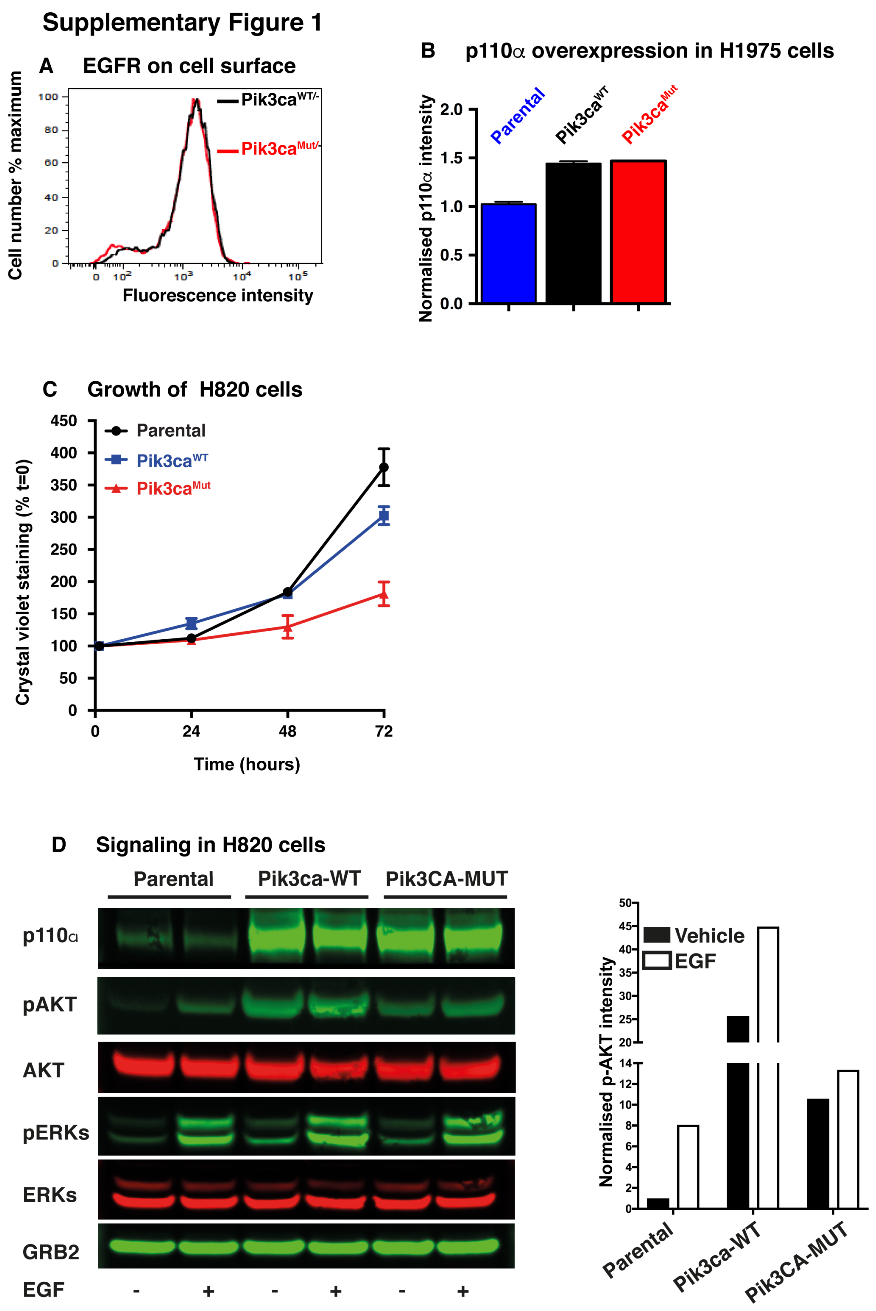

Supplementary Figure 1, relating to Figure 1. A. *Pik3ca*$^{WT/-}$ and *Pik3ca*$^{MUT/-}$ MEFs were starved overnight and subjected to FACs analysis to determine the amount of total EGFR on the cell surface. **B.** Quantification of the expression of p110α in H1975 cells (n=3) expressing either a WT or a MUT version of p110α, or parental H1975 cells. **C**. H820 EGFR mutant lung cancer cells were infected with retroviruses encoding Pik3ca^{WT}, Pik3ca^{MUT} or not infected (parental) and cell growth assays performed with crystal violet staining. **D.** H820 cells from (C) were then starved and stimulated with EGF (10 ng/ml) or vehicle for 10 minutes. Proteins were collected and subjected to Western Blot analysis as indicated.

Supplementary Figure 2

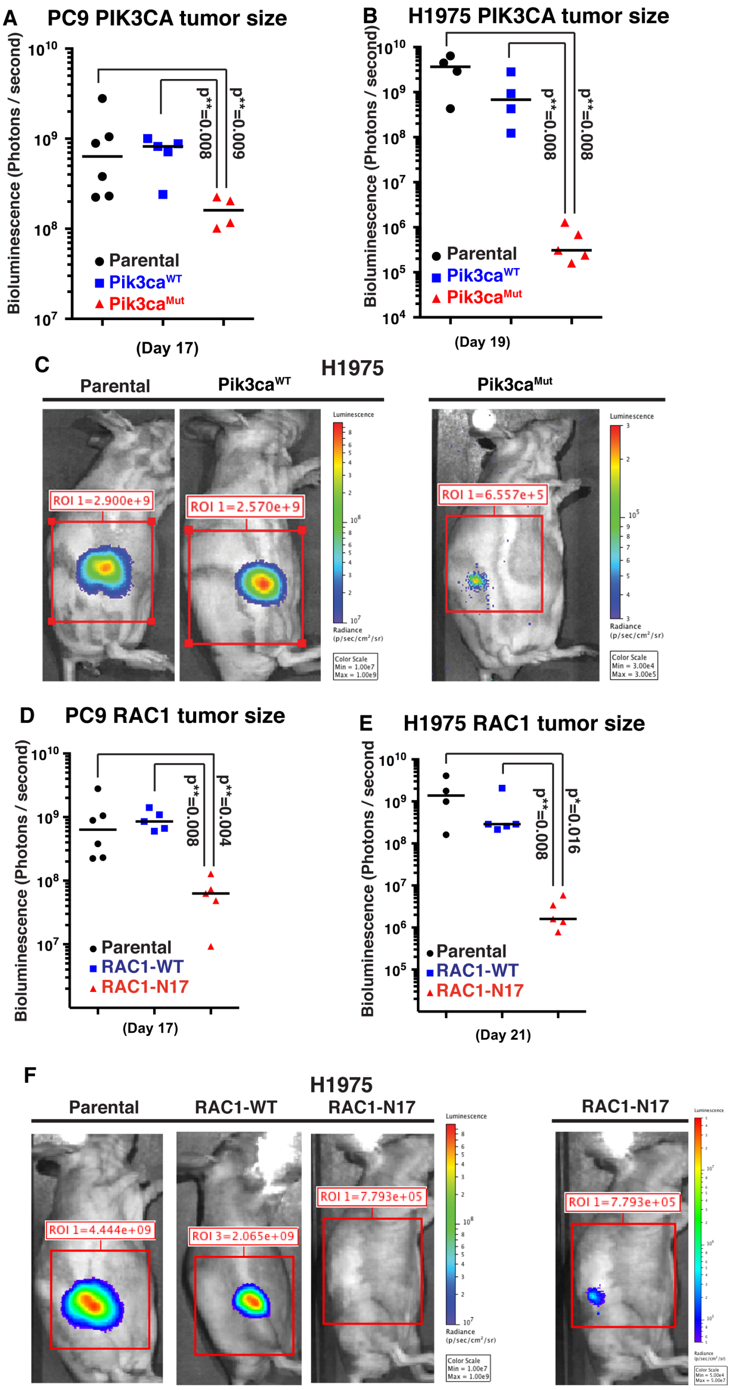

Supplementary Figure 2, relating to Figure 2. A and **B.** Quantification of bioluminescence emission in individual nude mice injected with PC9 or H1975 cells expressing Pik3ca^{WT} or Pik3ca^{MUT} at the end of the experiments from figures 2C (Parental PC9 n=6; PC9 Pik3caWT n=5; PC9 Pik3caMUT n=4) and 2D (Parental H1975 n=4; H1975 Pik3caWT n=4; H1975 Pik3caMUT n=5). **C.** Images of mice injected with H1975 parental cells, or cells expressing Pik3ca^{WT} or Pik3ca^{MUT}. For cells expressing Pik3ca^{MUT}, the scale was lowered to allow visualization (right panel). **D** and **E.** Quantification of bioluminescence emission in individual nude mice injected with PC9 or H1975 cells expressing Rac1WT or Rac1N17 at the end of the experiments from figures 2E (Parental PC9 n=6; PC9 Rac1WT n=5; PC9 Rac1N17 n=5) and 2F (Parental H1975 n=4; H1975 Rac1WT n=5; H1975 Rac1N17 n=5). **F.** Images of mice injected with H1975 cells expressing Rac1^{WT} or Rac1^{N17}, or parental cells. For cells expressing Rac1^{N17}, the scale was lowered to allow visualization (right panel).

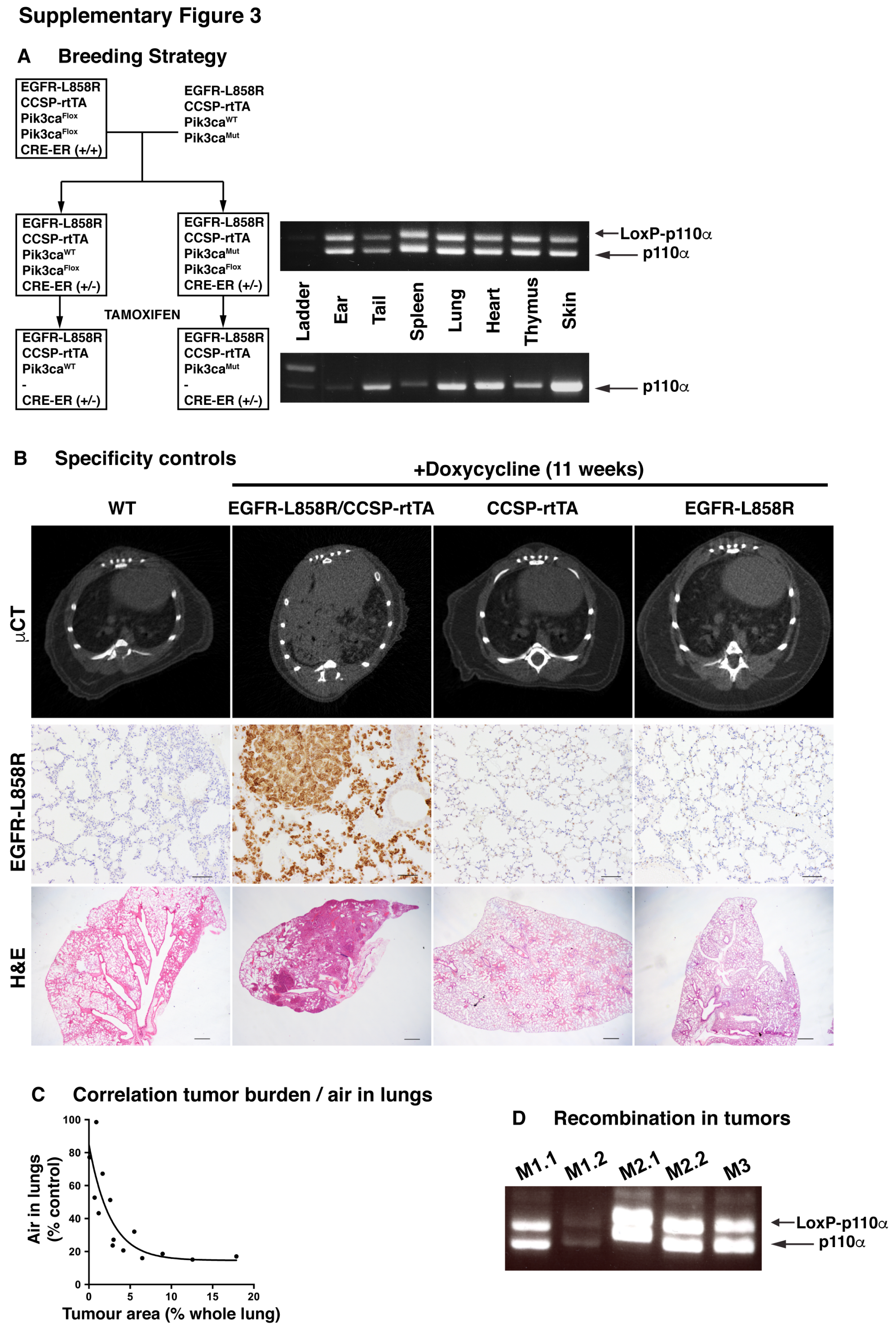

Supplementary Figure 3, relating to Figure 3. A. Mouse breeding strategy. Different tissues were analyzed before and after tamoxifen treatment to assess the deletion of the *Pik3ca*^{Flox} allele by PCR. Recombination data shown are for mice with *Pik3ca*^{Mut} allele. **B.** μCT (top line), EGFR^{L858R} staining (central line) and haematoxylin & eosin staining (lower line) of a wild type mouse (far left column), an EGFR^{L858R+}/CCSP-rtTA^{+} mouse (central-left column), an EGFR^{L858R-}/CCSP-rtTA^{+} mouse (central-right column) and a EGFR^{L858R+}/CCSP-rtTA^{+} mouse (far right column). **C.** Plot showing the correlation between air content in the lung measured by μCT versus tumor burden measured in histological samples (n=14). **D.** Tumor samples from EGFR^{L858R-} mice were harvested and analyzed to assess the deletion of the *Pik3ca*^{Flox} allele by PCR (n=5 from 3 different mice). Scale bars shown are 100μm.

Supplementary Figure 4

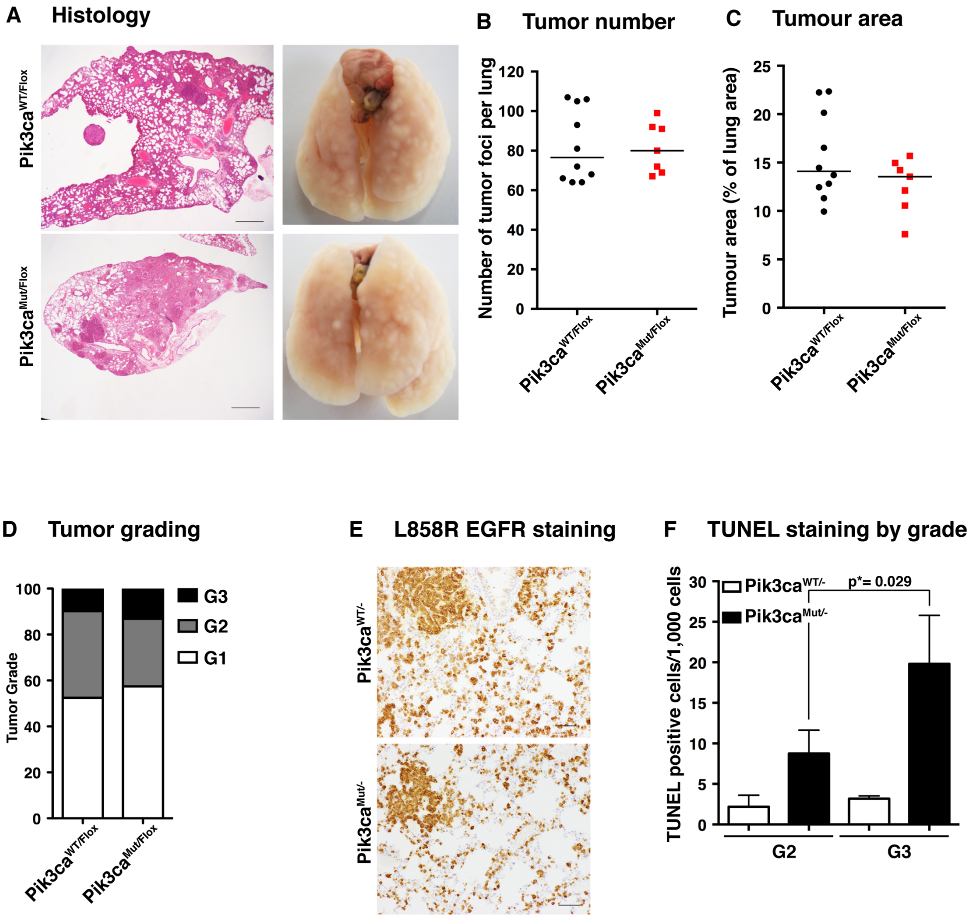

Supplementary Figure 4, relating to Figure 4. *Pik3ca*$^{WT/Flox}$ and *Pik3ca*$^{MUT/Flox}$ mice were fed with doxycycline-containing pellets for 11 weeks and sacrificed before tamoxifen treatment. **A.** Haematoxylin & eosin staining (left) of histological samples and images of ex-vivo lungs (right). **B.** Number of nodules quantified from histological samples. **C.** Representation of the area of the lung occupied by tumor foci quantified from histological samples. **D.** Distribution of tumor foci according to their grade in *Pik3ca*$^{WT/Flox}$ and *Pik3ca*$^{MUT/Flox}$ mice were fed with doxycycline-containing pellets for 11 weeks. In all cases, *Pik3ca*$^{WT/Flox}$ n=10 and *Pik3ca*$^{MUT/Flox}$ n=7. **E.** EGFR^{L858R} staining in histological samples after 5 days of tamoxifen treatment. **F.** Analysis of apoptosis by TUNEL staining segregated by tumor grade (*Pik3ca*$^{WT/-}$ G2 n=4; *Pik3ca*$^{WT/-}$ G3 n=3; *Pik3ca*$^{MUT/-}$ G2 n=4; *Pik3ca*$^{WT/-}$ G3 n=4). Scale bars shown are 100µm.
